# Abstracts from The International Conference on Medicinal Plants and Bioeconomy & the 1st Sino-CPLP Symposium on Natural Products and Biodiversity Resources

**DOI:** 10.1186/s13020-018-0213-x

**Published:** 2018-11-26

## A1 At Plant, From Lab Bench to Bedside: A Translational Approach

### Alberto C. P. Dias^1,2,3^

#### ^1^Centre of Molecular and Environmental Biology (CBMA)-University of Minho, Campus de Gualtar, 4710-057 Braga, Portugal; ^2^CITAB-UM, University of Minho; ^3^Centre of Biological Engineering (CEB), University of Minho

##### **Correspondence:** Alberto C. P. Dias - acpdias@bio.uminho.pt

*Chinese Medicine* 2018, **13(Suppl 3):**A1

Extracts, infusions, or other types of preparations from medicinal plants have been used since ancient times for the treatment of several diseases, in what is commonly designated as “folk medicine”. In recent years, science has proven that some plant extracts, fractions or specific compounds may have an important role as drug sources with relevant properties.

In this work, particular emphasis will be given to neuroprotection, anti-inflammatory and skin healing properties of specific plants extracts/compounds in in vitro as well as in vivo models. New approaches, including nanotechnology, will be addressed.

Based on specific extracts some particular topical formulations were developed, used in “real situations” for skin problems like psoriasis, pressure ulcers, and chronic wounds. The synergy of the properties of selected plant constituents, gave very positive results associated with a high degree of skin hydration, contributing to cell regeneration. In all cases, after repeated applications, notorious improvements or complete treatment were observed, without significant side effects.

**Acknowledgements:** This work was supported by national funds from FCT—Portuguese Foundation for Science and Technology, under the projects PTDC/AGR-ALI/105169/2008, PEst-OE/AGR/UI4033/2014, and INTERACT—ISAC project, no. NORTE-01-0145-FEDER-000017, co-financed by the European Regional Development Fund (ERDF) through NORTE 2020 (North Regional Operational Program 2014/2020).

## A2 Antioxidant and neuroprotective effects of Hyptis suaveolens, *Hyptis pectinata* and *Hyptis marrubioides* in Caenorhabditis elegans

### Daniela Vilas-Boas Campos^1,2,3^, Rejaine Rios^3,4^, Carlos Bessa^1,2^, Marta Daniela Costa^1,2^, Andreia Teixeira-Castro^1,2^, Patrícia Maciel^1,2^, Alberto C. P. Dias^3,5,6^

#### ^1^Life and Health Sciences Research Institute (ICVS), School of Medicine, University of Minho, Campus Gualtar, 4710-057 Braga, Portugal; ^2^ICVS/3B’s-PT Government Associate Laboratory, Braga/Guimarães, Portugal; ^3^Centre of Molecular and Environmental Biology (CBMA)-University of Minho, Campus de Gualtar, 4710-057 Braga, Portugal; ^4^Instituto Federal Goiano, Biology Departament, Campus Rio Verde, Goiás, Brasil; ^5^CITAB-UM, University of Minho, Portugal; ^6^Centre of Biological Engineering (CEB), University of Minho

##### **Correspondence:** Alberto C. P. Dias - acpdias@bio.uminho.pt

*Chinese Medicine* 2018, **13(Suppl 3):**A2

The increasing trend for the use of natural products as sources of pharmacologically active molecules has changed attitudes in the population. Given the existing demand, a credible scientific analysis and validation of the effect of these natural products is necessary. The genus Hyptis Jacq. (Lamiaceae) has about 300 species with wide distribution, among which *Hyptis suaveolens*, *Hyptis pectinata* and *Hyptis marrubioides* (HS, HP, HM, respectively) are used in folk medicine and are commercialized in street markets for treatment of several diseases. This study aims to evaluate the neuroprotective activity, as well as to elucidate some of the cellular mechanisms involved in the pharmacological action of HS/HP/HM plant extracts using *Caenorhabditis elegans*, as an animal model. For this purpose, we used a *C. elegans* model of Machado-Joseph disease (MJD), expressing a human mutant ATXN-3 and a *C. elegans* model of frontotemporal dementia with parkinsonism-17 (FTDP-17), expressing a mutant form of tau protein, and tested ethanolic leaf extracts from HS, HP, and HM. Our data showed that chronic treatment with 1 mg/mL of HS/HP/HM extracts had a beneficial impact in these diseases since it significantly ameliorated the locomotor defects exhibited by *C. elegans*. Moreover, with C. elegans model of MJD, the chronic treatment with the Hyptis extracts also increased the animal’s survival. We observed, in both models, a significant protection against juglone-induced oxidative damage (by more than 50%), after chronic treatment with these extracts. Using *C. elegans* reporter strains we also observed a higher induction of gst-4, in HS/HP/HM extract-treated animals upon exposure to oxidative damage. Our findings support an antioxidant and neuroprotective activity of HS, HP e HM, suggesting the activating specific antioxidant enzymes like gst-4.

## A3 Preparative-HPLC–MS technique for rapid isolation of a chemical constituent from *Helichrysum odoratissimum*

### Weiyang Chen^1^, Sushil K. Chaudhary^1^, Khotso D. Serabele^1^, Sandra Combrinck^1,2^, Alvaro M. Viljoen^1,2^

#### ^1^Department of Pharmaceutical Sciences; ^2^SAMRC Herbal Drugs Research Unit, Tshwane University of Technology, 175 Nelson Mandela Drive, Private Bag X680, Pretoria 0001, South Africa

##### **Correspondence:** Weiyang Chen - chenw@tut.ac.za

*Chinese Medicine* 2018, **13(Suppl 3):**A3

**Background:** *Helichrysum Odoratissimum* is commonly used as a traditional medicine to relieve abdominal pain heartburn, cough and colds, and to treat wound [1]. The flavonoids, phloroglucinols, α-pyrones, coumarins and terpenoids have been found in several *Helichrysum Odoratissimum* species. Preparative (prep) HPLC is a powerful tool for the isolation of compounds with excellent efficiency and high recovery [2]. Combined with mass spectroscopy, prep HPLC has become a powerful tool in the pharmaceutical industry for the targeted isolation of biological molecules and drugs from natural products.

**Fig. 1:**
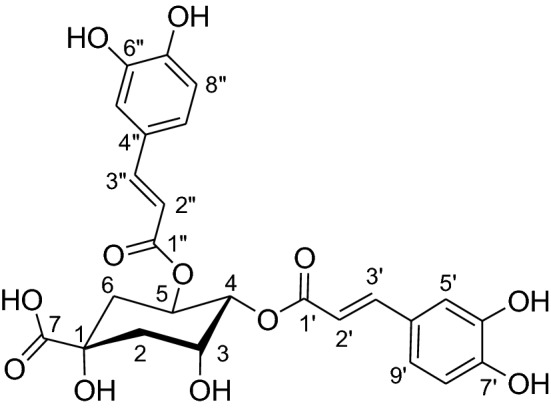
Structure of 4,5-dicaffeoylquinic acid

**Results and discussion:** The purity of this isolate was determined as 99.9% using UPLC-PDA. The compound was identified as 4,5-dicaffeoylquinic acid (Fig. 1) [3,4], using a combination of UV spectrophotometry, UPLC-MS and nuclear magnetic resonance (1H-NMR) spectroscopy. 4,5-Dicaffeoylquinic acid is known to possess good antioxidative, tyrosinase inhibitory, antiproliferation and anti-HIV activities. The compound has not been previously identified in *H. odoratissimum.*

**Conclusion:** Prep-HPLC–MS was found to be a useful tool for the direct isolation of 4,5-dicaffeoylquinic acid from the crude extract. The compound can serve as a marker compound for quality control of this important species.

**Acknowledgement:** The work was financially supported by the SAMRC Herbal Drugs Research Unit, South Africa.

**References**

1. Lourens ACU, Viljoen AM, van Heerden FR. South African *Helichrysum* species: a review of the traditional uses, biological activity and phytochemistry. J Ethnopharmacol. 2008; 119:630–52.
2. Unger KK. Handbuch der HPLC, Darmstadt: GIT Verlag; 1994.
3. Farah A, Monteiro M, Donangelo CM. Lafay Chlorogenic acids from green coffee extract are highly bioavailable in humans. S J Nutr. 2008; 138:2309–15.
4. Heyman HM, Senejoux F, Seibert I, Klimkait T, Maharaj VJ, Meyer JJM. Identification of anti-HIV active dicaffeoylquinic- and tricaffeoylquinic acids in *Helichrysum populifolium* by NMR-based metabolomic guided fractionation. Fitoterapia 2015; 103:155–64.

## A4 Exploring the bioactivities of *Thymus* species extracts in search of new sources of nutraceuticals

### Amélia M. Silva^1^, Carlos Martins-Gomes^1^, Luis M. Félix^1^, Meriem Taghouti^1^, Eliana B. Souto^2^, João A. Santos^3^, Fernando M. Nunes^4^

#### ^1^Department of Biology and Environment (DeBA-ECVA); University of Trás-os-Montes e Alto Douro (UTAD); Quinta de Prados; 5001-801 Vila Real, Portugal; ^2^Department of Pharmaceutical Technology; Faculty of Pharmacy, University of Coimbra, Pólo das Ciências da Saúde, Azinhaga de Santa Comba, 3000-548 Coimbra, Portugal; ^3^Department of Physics (DF-ECT), UTAD, 5001-801 Vila Real, Portugal; ^4^Department of Chemistry (DQ-ECVA), UTAD; Quinta de Prados, 5001-801 Vila Real, Portugal

##### **Correspondence:** Amélia M. Silva - amsilva@utad.pt

*Chinese Medicine* 2018, **13(Suppl 3):**A4

**Background:** Among the aromatic and medicinal plants, Lamiaceae family has gained huge interest as it comprises several plants used worldwide as condiments and as medicinal preparations, as is the case of *Thymus* genus. This genus comprises more than 300 species, being some endemic to the Mediterranean region, and a few endemic to Portugal.

We have been studying several *Thymus* species, comparing some that are already part of human diet with others not yet introduced into the diet, aiming to evaluate their potential use as medicinal and functional foods. The study includes *T. carnosus*, *T. fragrantissimus*, *T. pulegioides*, *T. vulgaris* species, and others.

**Materials and methods:** Extracts were prepared with water (decoction and infusion) and hydroethanolic (20:80, v/v) mixture. Chemical composition was characterized using colorimetric and HPLC methods. Bioactivities relevant to human health (e.g. antioxidant, anti-proliferative, anti-inflammatory, antidiabetic and neuroprotective) were evaluated [1, 2].

**Results:** Most extracts present as main component rosmarinic acid (~ 48% of total identified phenols; *T. pulegioides* hydroethanolic extracts [1]) while *T. carnosus* presents Salvianolic acid K and A as main components (~ 76% of total identified phenols in hydroethanolic extracts [2]). All extracts presented good antioxidant and radical scavenging activities as well as dose- and time-dependent anti-proliferative activities against tumoral cell lines (e.g. Caco-2, HepG2, RAW 264.7). *T. carnosus* showed good anti-inflammatory activity (~ 80% NO release inhibition by LPS-stimulated RAW 264.7 cells with 200 µg/mL decoction). *T. pulegiodes* showed high neuroprotective activity (80% and 94% acetylcholinesterase and tyrosinase inhibition, respectively). *T. fragrantissimus* revealed good anti-aging activity (~ 50% elastase inhibition, at 1 mg/mL). All extracts showed poor anti-diabetic activities.

**Conclusions:** Our study highlights the important potential of these *Thymus* species as functional food ingredients and as potential sources of molecules for pharmaceutical and cosmeceutical industries.

**Acknowledgements**: Financed by INTERACT-project-NORTE-01-0145-FEDER-000017, ISAC, co-financed by ERDF through NORTE2020, (BI/UTAD/INTERACT/ISAC/203/2016, to L.F.); Ervital, FCT (grant to M.T.(PD/BD/52563/2014).

**References**

1. Martins-Gomes C, Taghouti M, Schäfer J, Bunzel M, Silva AM, Nunes FM. Chemical characterization and bioactive properties of decoctions and hydroethanolic extracts of *Thymus carnosus* Boiss. J Funct Foods. 2018; 43:154–64.
2. Taghouti M, Martins-Gomes C, Schäfer J, Félix LM, Santos JA, Bunzel M, Nunes FM, Silva AM. *Thymus pulegioides* L. as a rich source of antioxidant, anti-proliferative and neuroprotective phenolic compounds. Food and Funct. 2018; 9(7):3617–29.

## A5 Flow cytometry study of cell cycle, apoptosis and oxidative stress of *Thymus* derived extracts in human colorectal cancer cell line (Caco-2)

### Luis M. Félix, Tiago E. Coutinho, Carlos Martins-Gomes, Amélia M. Silva

#### Department of Biology and Environment (DeBA-ECVA); University of Trás-os-Montes e Alto Douro (UTAD); Quinta de Prados; 5001-801 Vila Real, Portugal

##### **Correspondence:** Amélia M. Silva - amsilva@utad.pt

*Chinese Medicine* 2018, **13(Suppl 3):**A5

**Background:** Plant extracts have been used in traditional medicine for prevention and treatment of several pathologies. In particular, *Thymus* genus (Lamiaceae family) has been used traditionally due to the long list of recognized pharmacological properties which have been linked to their phenolic composition. Among *Thymus* species, *Thymus vulgaris* has been extensively studied contrarily to *Thymus vulgaris* variety *fragantissimus*, a citrusy orange peel scent and flavor, that remains one of the least studied, with very little information related to its effects on human colorectal cancer cell line (Caco-2). In this work, the effects of hydroethanolic (HE) and aqueous extracts (decoction and infusion) from both plant species were investigated regarding their role in basal oxidative stress modulation.

**Materials and methods:** Oxidative stress was assessed by measuring reactive oxygen species (ROS), glutathione levels, lipid peroxidation and mitochondrial membrane potential. Cell cycle and apoptosis induction were also evaluated. All assays were performed by flow cytometry using adequate fluorescent probes.

**Results:** Cells were exposed to the 0%, 10%, 50%, and 90% inhibitory concentrations (Control, IC10, IC50, and IC90, respectively) for 24-h. HE extracts from both species induced changes in the percentage of living cells mediated by an increase in cellular ROS levels (notorious to infusions and decoctions of *T. fragrantissimus*), accompanied by increase in cytosolic glutathione levels (notorious to HE of *T. fragrantissimus*). Dose-dependent lipid damage was observed, particularly to higher concentrations, but mitochondrial membrane potential was not affected. Cell cycle arrest was detected, to all extracts. Effects of infusion extracts were less notorious, comparing to the remaining extracts, in all assays.

**Conclusion:** These extracts have possible therapeutic effects on Caco-2 cells (colorectal cancer cell model) and its components can be suitable candidates for the development of new drugs.

**Acknowledgment:** INTERACT-project–NORTE-01-0145-FEDER-000017 (ISAC), co-financed by ERDF through NORTE-2020 and grants (BI/UTAD/INTERACT/ISAC/203/2016, to L.F.).

## **A6***Cucurbita ficifolia* Bouché extracts promotes ROS scavenging activity and cell cycle arrest

### Tiago E. Coutinho^1^, Tiago Monteiro^1^, Fernando M. Nunes^2^, Amélia M. Silva^1^

#### ^1^Department of Biology and Environment (DeBA-ECVA), University of Trás-os-Montes e Alto Douro; Quinta de Prados, 5001-801 Vila Real, Portugal; ^2^Department of Chemistry (DQ-ECVA), University of Trás-os-Montes e Alto Douro; Quinta de Prados, 5001-801 Vila Real, Portugal

##### **Correspondence:** Amélia M Silva - amsilva@utad.pt

*Chinese Medicine* 2018, **13(Suppl 3):**A6

**Background:** *Cucurbita ficifolia* Bouché (chila pumpkin) is a type of squash native from México, being used for human nutrition especially in crystallized pastry and deserts and also as medical purposes, such as for the treatment of diabetes type 2, in wound-healing and for fever. There are a few ethnopharmacological studies reporting its usage as well as some studies performed in which a hypoglycemic effect was demonstrated in animal models fed with this pumpkin, but there is a lack of information concerning its phytochemical profile as well as of other bioactivities.

**Materials and methods:** We have performed hydroethanolic ((20:80) %v/v) extracts, from pulp and from peel, that were further fractionated into methanolic (H.E-Fr.MeOH) and aqueous fraction (H.E-Fr.Aq). The phenolic profile was determined and the extracts were screened for biological activities. Cell viability was assessed by Alamar Blue, and intracellular ROS were assessed by flow cytometry.

**Results:** Extracts and fractions shown to be rich in polyphenolic compounds and in sugars. Extracts exerted a moderate anti-proliferative activity against Caco-2 cells (dose- and time-dependent). Using flow cytometry and DCFDA probe (2′,7′-Dichlorofluorescein diacetate) we evaluate the Caco-2 cells content in reactive oxygen species (ROS) after being exposed to different concentrations of extracts in the absence and in the presence of H_2_O_2_ (ROS inducer). Pre-exposure of Caco-2 cells to aqueous fraction of pulp extract protected the cells against ROS (induced by H_2_O_2_ exposure), but when cells were pre-exposed to the methanolic fractions this protection was not observed. We also have observed that methanolic extracts dose-dependently increased intracellular ROS and modulated the cell cycle in respect to control cells.

**Conclusions:** consumption of *Cucurbita ficifolia* pulp has a positive effect concerning to ROS protection and has a moderate role as anti-cancer agent against colorectal carcinoma cells (Caco-2 cells).

**Acknowledgements:** INTERACT-project–NORTE-01-0145-FEDER-000017, ISAC, co-financed by ERDF through NORTE2020.

## A7 Eriodyctiol and Quercetin radical scavenging, anti-proliferative and anti-inflammatory activity: a comparative study

### Tiago Monteiro, Carlos Martins-Gomes, Dario Santos, Amélia M. Silva

#### Department of Biology and Environment (DeBA-ECVA), University of Trás-os-Montes e Alto Douro (UTAD), Quinta de Prados, 5001-801 Vila Real, Portugal

##### **Correspondence:** Amélia M. Silva - amsilva@utad.pt

*Chinese Medicine* 2018, **13(Suppl 3):**A7

**Background:** Plants are used for medicinal purposes worldwide. Although there is still a large gap of knowledge between the therapeutic effects attributed to medicinal plants and the underlying cellular mechanisms. Aiming the clarification of such mechanisms it is important to know plant’s composition and the bioactivities of different compounds. One of the main classes of compounds present in the Plantae kingdom is the flavonoids. These phytochemicals are related to a wide variety of bioactivities such antioxidant and anti-inflammatory. In this work we aimed to study some bioactivities of eriodyctiol and to compare with quercetin.

**Materials and methods:** Cell viability and anti-proliferative activity was assessed in HepG2 and RAW 264.7 cell lines exposed for 24 and 48 h to different doses of the compounds, using Alamar Blue method. Anti-inflammatory activity was evaluated by inhibition of NO radical, both using a radical scavenging assay and using a cellular model and Griess reagent [1]. Radical scavenging assays were performed by colorimetric methods [1]. Flow cytometry was used to assess oxidative stress.

**Results:** We have observed a decrease in cell viability, dose- and time-dependent, with identical pattern for both flavonoids. ABTS assay revealed dose-dependent increase of antioxidant activity, with quercetin showing higher activity. Antioxidant effect of eriodyctiol and quercetin was confirmed in HepG2 cells, by flow cytometry with ROS probe DCFDA. Concerning NO scavenging activity, dose-dependent effect was observed and quercetin revealed to be more effective. Using LPS-stimulated RAW 264.7 cells, both eriodyctiol and quercetin induced equal inhibition of NO release from RAW 264.7 cells.

**Conclusions:** Comparing to quercetin, a very well-studied flavonoid, eriodyctiol has good antioxidant and anti-inflammatory potential.

**Acknowledgements:** INTERACT-project–NORTE-01-0145-FEDER-000017, ISAC, co-financed by ERDF through NORTE2020.

**References**

1. Martins-Gomes C, Taghouti M, Schäfer J, Bunzel M, Silva AM, Nunes FM. Chemical characterization and bioactive properties of decoctions and hydroethanolic extracts of *Thymus carnosus* Boiss. J Funct Foods. 2018; 43:154–64.

## A8 Vitamin E isoform γ-Tocotrienol: A potential remedy for asthma and COPD

### W. S. Fred Wong^1,2,3^, Hong Yong Peh^1^

#### ^1^Department of Pharmacology, Yong Loo Lin School of Medicine, National University Health System, Singapore; ^2^Immunology Program, Life Science Institute, National University of Singapore, Singapore; ^3^Singapore-HUJ Alliance for Research and Enterprise (SHARE), Molecular Mechanisms of Inflammatory Diseases (MMID) Program, National University of Singapore, Singapore

##### **Correspondence:** W. S. Fred Wong - phcwongf@nus.edu.sg

*Chinese Medicine* 2018, **13(Suppl 3):**A8

**Background:** Inflammation and oxidative damage contribute to the pathogenesis of asthma and COPD. As vitamin E isoform γ-tocotrienol possesses both anti-oxidative and anti-inflammatory properties, we aimed to establish the therapeutic potential of γ-tocotrienol in a house dust mite (HDM)-induced mouse asthma model and a cigarette smoke-induced COPD models.

**Materials and methods:** For asthma, BALB/c mice were sensitized and challenged with HDM. For COPD, BALB/c mice were exposed to cigarette smoke (CS) daily for 2 months. Bronchoalveolar lavage (BAL) fluid and lung tissues were assessed for cell infiltration and mucus hypersecretion, oxidative damage, and regulation of transcription factor activities. Airway hyperresponsiveness (AHR) in response to methacholine, and lung function parameters were measured.

**Results:** γ-Tocotrienol displayed better free-radical neutralizing activity in vitro and inhibition of BAL fluid total, eosinophil and neutrophil counts in HDM mouse asthma in vivo. γ-Tocotrienol abated HDM-induced elevation of BAL fluid cytokines and chemokines, total reactive oxygen species and oxidative damage biomarkers, and of serum IgE levels, but promoted lung endogenous antioxidant activities. γ-Tocotrienol markedly suppressed methacholine-induced AHR in mouse asthma. On the other hand, γ-tocotrienol reduced CS-induced BAL fluid neutrophil count and levels of cytokines, chemokines and oxidative damage biomarkers, and restored lung endogenous antioxidant activities. Mechanistically, γ-tocotrienol blocked nuclear STAT3 and NF-κB levels and enhanced nuclear Nrf2 levels in the lungs. γ-Tocotrienol ameliorated bronchial epithelium thickening and destruction of alveolar sacs in lungs, and improved lung functions.

**Conclusions:** We revealed for the first time the anti-inflammatory and antioxidant efficacies of vitamin E isoform γ-tocotrienol in allergic asthma and COPD models. In addition, γ-tocotrienol was able to attenuate emphysematous lesions and improve lung function in COPD. γ-Tocotrienol may have therapeutic potential for the treatment of asthma and COPD.

**Acknowledgements:** This work was supported in part by NMRC/CBRG/0027/2012 from the NMRC of Singapore and the NRF grant R-184-000-269-592 from the National Research Foundation of Singapore.

## A9 Crotalicidin, cathelicidin-related peptide from rattlesnake venom gland, a template for production of oligopeptide fragments with broad spectrum of antiproliferative activities

### Gandhi Rádis-Baptista

#### Laboratory of Biochemistry and Biotechnology, Federal University of Ceará, Fortaleza-CE, Brazil

##### **Correspondence:** Gandhi Rádis-Baptista - gandhi.radis@ufc.br

*Chinese Medicine* 2018, **13(Suppl 3):**A9

Crotalicidin (Ctn), a 34 amino acid-long, linear alpha-helical peptide that belongs to the group of vipericidins and to the cathelicidin family of vertebrate antimicrobial peptides, was characterized from the venom gland of the *Crotalus durissus terrificus* (South American rattlesnake) and its antiproliferative activity was demonstrated against bacteria and cancer cells [1]. The activity of Ctn against trypanosomatid protozoa was also experimentally evidenced in vitro [2]. The Ctn was structurally dissected in two pharmacophore fragments, the 14-residue, N-terminal Ctn[1–14] and the 20-residue, C-terminal Ctn[15–34], retrieving the antimicrobial and antitumor activities of the full-length peptides [3]. In comparison to the full-length Ctn, the N- and C-terminal fragments showed distinct and selective activity against Gram-negative bacteria and some lines of tumor cells. Against Gram-negative bacteria, such as *Escherichia coli* and *Pseudomonas aeruginosa*, the antibacterial activity of Ctn[15–34] involves membrane perturbation, cytoplasmic internalization and interaction with nucleic acids [4]. The antifungal effects of these peptides were also demonstrated against amphotericin B-susceptible isolates of pathogenic yeasts and it was shown that the underlying mechanism by which Ctn[15–34] kills such eukaryotic pathogen involves membrane disruption and induction of early apoptosis and late necrosis [5]. Ctn [15–34] was also effective in vitro as an antiviral agent against infection myonecrosis virus (IMNV), a RNA virus that affects farming shrimps [6]. Interestingly, repetitive in tandem encrypted vipericidin peptides display selective structure-related cytotoxicity to zebrafish and mammary gland tumor cells [7]. Altogether, the broad spectrum of activities of these venom- and cathelicidin-derived peptide compounds, the antiproliferative activity is amenable to be expanded against selected targets and the core peptide structure tamed to be developed in innovative pharmaceutical products.

**References**

1. Falcao CB, de La Torre BG, Perez-Peinado C, Barron AE, Andreu D, Radis-Baptista G. Vipericidins: a novel family of cathelicidin-related peptides from the venom gland of South American pit vipers. Amino acids. 2014;46(11):2561–71.
2. Bandeira ICJ, Bandeira-Lima D, Mello CP, Pereira TP, De Menezes R, Sampaio TL, et al. Antichagasic effect of crotalicidin, a cathelicidin-like vipericidin, found in Crotalus durissus terrificus rattlesnake’s venom gland. Parasitology. 2017:1–6.
3. Falcao CB, Perez-Peinado C, de la Torre BG, Mayol X, Zamora-Carreras H, Jimenez MA, et al. Structural Dissection of Crotalicidin, a Rattlesnake Venom Cathelicidin, Retrieves a Fragment with Antimicrobial and Antitumor Activity. J Med Chem. 2015;58(21):8553–63.
4. Perez-Peinado C, Dias SA, Domingues MM, Benfield AH, Freire JM, Radis-Baptista G, et al. Mechanisms of bacterial membrane permeabilization by crotalicidin (Ctn) and its fragment Ctn(15–34), antimicrobial peptides from rattlesnake venom. The Journal of biological chemistry. 2018;293(5):1536-49.
5. Cavalcante CSP, de Aguiar FLL, Fontenelle ROS, de Menezes R, Martins AMC, Falcao CB, et al. Insights into the candidacidal mechanism of Ctn[15-34]—a carboxyl-terminal, crotalicidin-derived peptide related to cathelicidins. J Med Microbiol. 2018;67(1):129–38.
6. Vieira-Girao PRN, Falcao CB, Rocha I, Lucena HMR, Costa FHF, Radis-Baptista G. Antiviral Activity of Ctn[15-34], A Cathelicidin-Derived Eicosapeptide, Against Infectious Myonecrosis Virus in Litopenaeus vannamei Primary Hemocyte Cultures. Food Environ Virol. 2017;9(3):277–86.
7. Wang L, Chan JY, Rego JV, Chong CM, Ai N, Falcao CB, et al. Rhodamine B-conjugated encrypted vipericidin nonapeptide is a potent toxin to zebrafish and associated with in vitro cytotoxicity. Biochimica et biophysica acta. 2015;1850(6):1253–60.

## A10 Isolation and characterization of *Acanthus ilicifolius* mitochondrial proteins: A Proteomic and Bioinformatics approach

### Ganesh Sekaran^1,2^, Jannet Vennila J^3^, Xiaoying Zhang^1,4^

#### ^1^College of Veterinary Medicine, Northwest Agriculture and Forestry University, Yangling, Shaanxi, 712100, P. R. China; ^2^Department of Biotechnology, Nehru Arts and Science College, T.M. Palayam, Coimbatore, 641105, Tamil Nadu, India; ^3^Department of Agriculture, Karunya Institute of Technology & Sciences, Coimbatore, 641 114, Tamil Nadu, India; ^4^Centre of Molecular and Environmental Biology, University of Minho, Department of Biology Campus de Gualtar, Braga, Portugal

##### **Correspondence:** Xiaoying Zhang - zhang.xy@nwsuaf.edu.cn; zhxying@yahoo.com

*Chinese Medicine* 2018, **13(Suppl 3):**A10

**Background information:** *Acanthus ilicifoilus* have been reported to have important medicinal properties used in traditional Indian and Chinese Medicine. Proteins contribute to the medicinal properties and salt resistance properties of this plant. Until now, no detailed studies have been reported to identify the novel proteins or to augment their role in the metabolism of this plant.

**Purpose:** The genome of the plant is not sequenced. With the limited information an attempt was made to identify the proteins located in the mitochondria available from other plant resources.

**Methods:** The mitochondria were isolated from *A. ilicifolius* leaves by Percoll density gradient centrifugation. The *A. ilicifolius* mitochondrial proteins were systematically separated by 2D-PAGE following by CBB and silver staining. Totally 151 protein spots visualized from pI 3-10 and pI 4-7. The gel was analyzed by Image Master. The pI and Mw of each protein spots were compared with plant database using a Python program. Three spots were analyzed by MALDI-TOF/MS.

**Results and conclusions:** Among the 151 proteins identified from 2D-PAGE, 27 spots were matched with known proteins and 23 with unknown proteins of mitochondrial location. The identified proteins were found to be in an involved in TCA cycle, Electron Transport Chain, Transport mechanism and Amino acid synthesis of mitochondria. Protein–protein interaction was analyzed using Hex 6.0 Cuda software. Modeled structures were retrieved from PDB (ligand) against salt stress proteins (receptor). Halo acid Dehalogenase-like hydrolase (HAD) and Triose Phosphate isomerase from *A. ilicifolius* proteins show highest interaction with Desiccation-related protein PCC13-62, Phosphatidylinositol 4-phosphate 5-kinase 1, Aquaporin TIP2-3 and HVA22-like protein b which was responsible for the salt stress and hygroscopic related proteins. MALDI-TOF/MS results were matched with *Arabidopsis thaliana* and *Coptis japonica*. Proteins details were submitted in World-2D PAGE Repository.

**Acknowledgements:** This work was supported by the National Key Research and Development Program of China (2017YFD0501400) and the Key Constructions Program (2015SD0018) of International Cooperation Base in S&T, Shaanxi, China, as well as the strategic programme UID/BIA/04050/2013 (POCI-01-0145-FEDER-007569) funded by national funds through the FCT I.P., by the Ministério da Ciência, Tecnologia e Ensino Superior (MCTES) and by the ERDF through the COMPETE2020—Programa Operacional Competitividade e Internacionalização (POCI), Portugal.

